# Balance and prospective falls in patients with rheumatoid arthritis

**DOI:** 10.1186/s12891-022-05489-1

**Published:** 2022-06-07

**Authors:** Sabine Wiegmann, Gabriele Armbrecht, Diana Borucki, Bjoern Buehring, Frank Buttgereit, Christian Detzer, Désirée Schaumburg, Kim Nikola Zeiner, Roswitha Dietzel

**Affiliations:** 1 Department of Radiology, Centre for Muscle and Bone Research, Charité – Universitätsmedizin Berlin, corporate member of Freie Universität Berlin and Humboldt- Universität zu Berlin, Hindenburgdamm 30, 12200 Berlin, Germany; 2 Deutsche Rheuma-Liga Bundesverband e.V., Welschnonnenstraße 7, 53111 Bonn, Germany; 3 Bergisches Rheuma-Zentrum, Krankenhaus St. Josef, Bergstr. 6-12, 42105 Wuppertal, Germany; 4 Department of Rheumatology and Clinical Immunology, Charité – Universitätsmedizin Berlin, corporate member of Freie Universität Berlin and Humboldt- Universität zu Berlin, Charitéplatz 1, 10117 Berlin, Germany; 5 Department of Dermatology, Venereology and Allergology, Goethe University Frankfurt, Theodor-Stern-Kai 7, 60596 Frankfurt/Main, Germany

**Keywords:** Rheumatoid arthritis, Fall, Balance, Postural sway, Postural control, One-leg stand

## Abstract

**Background:**

Postural control is associated with fall risk. Patients with rheumatoid arthritis (RA) have a higher risk to fall than healthy subjects. The objective of this study was to identify associations between variables of postural control with prospective falls in patients with RA.

**Methods:**

For the baseline, the balance performance of 289 men and women with RA, ages 24–85 years, was evaluated by SPPB, FICSIT-4 and Romberg tests. Postural sway for Romberg, semitandem, tandem and one-leg stands were measured with the Leonardo Mechanograph®. Self-reported disability was assessed using the Health Assessment Questionnaire (HAQ) and the Activity-specific Balance Confidence Scale (ABC-scale). Falls were reported in quarterly reports over a year. Univariate and multiple logistic regression analysis were used to explore any associations with falling. Receiver-operating characteristics were determined, and the area under the curve is reported.

**Results:**

A total of 238 subjects completed the 1-year follow-up, 48 (20.2%) experienced at least one fall during the observational period. Age (OR = 1.04, CI 1.01–1.07), HAQ (OR = 1.62, 1.1–2.38), FICSIT-4 scoring 0–4 (OR = 2.38, 1.13–5.0), and one-leg standing (OR = 2.14, 1.06–4.31) showed significant associations with falls. With regard to the SPPB and ABC-scale, no statistically significant associations with falls were found. The quartiles containing the worst results of medio-lateral sway of Romberg (OR = 2.63, CI 1.03–6.69), total sway of semitandem (OR = 3.07, CI 1.10–8.57) and tandem (OR = 2.86, CI 1.06–7.69), and area of sway of semitandem (OR = 2.80, CI 1.11–7.08) stands were associated with falls.

**Conclusions:**

The assessment of a one-leg stand seems to be a good screening tool to discriminate between high and low risk of falls in RA patients in clinical practice. A low FICSIT-4 score and several sway parameters are important predictors of falls.

**Trial registration:**

The study has been registered at the German Clinical Trials Register and the WHO International Clinical Trials Registry Platform (ICTRP) since 16 March 2017 (DRKS00011873).

## Key points

- The use of the one-leg stand as a screening tool allows a more precise discrimination between high and low risk of fall than SPPB in patients with RA.
- A low FICSIT-4 score identifies RA patients at high risk of falling.
- The medio-lateral sway of the Romberg and semitandem, the area of sway in semitandem, as well as the total path length and velocity of sway in semitandem and tandem stands can be used to predict falls.
- The utilisation of FICSIT-4 is superior to Romberg tests in research projects due to a sum score that compensates for missing values.
- The training of medio-lateral and anterior–posterior balance strategies should be the focus of fall prevention strategies and the therapy of fallers.

## Background

Balance is a multidimensional muscle function domain that is fundamentally related to preserving independence and mobility, and is one of the most important indicators for predicting falls [1, 2]. Postural control is defined as the ability to achieve or restore a state of balance during any posture or activity with a minimum of postural sway [3]. Poor postural control is related to an increased postural sway, resulting in a higher fall risk in healthy populations [2, 4, 5].

Patients with rheumatoid arthritis (RA) are at a higher risk of falling than healthy people [6, 7]. Known risk factors in patients with RA are a long duration of disease, high disease activity, medication and foot deformities [8–10]. Reduced postural control in patients with RA can be driven by structural changes such as painful, swollen or deformed joints, as well as a generale decline of muscle function [9]. Additionally, chronic inflammatory processes and the side effects of medication can reduce sensory input and neuromuscular responses, leading to reduced balance and increased risk of falls [11, 12]. The incidence of falls in populations with RA ranges from 36 to 50% in prospective studies [7, 12–14], compared to healthy older samples where it ranges between 6 and 34% [10, 15].

One of the most commonly used performance measures to screen for fall risk is the Short Physical Performance Battery (SPPB). The SPPB evaluates the results of a balance assessment (Romberg, Semitandem, Tandem), the gait speed and chair rise test with the highest score indicating high functional performance [16]. The instrument is considered reliable and valid in predicting falls [17, 18]. Another performance measure is the FICSIT-4 (Frailty and Injuries: Cooperative Studies of Intervention Technique-4), which is used to assess a Romberg, semitandem and tandem stance and, in contrast to the SPPB, additionally a one-leg stance [19]. To the best of the authors knowledge, in no prior studies has the FICSIT-4 been investigated with regard to the evaluation of fall risk in rheumatoid patients. The one-leg standing assessment is considered a predictor of falls [20]. Yet it is often not recommended, as it is too demanding for many patient populations [21]. However, integrating the one-leg stance into a balance assessment of patients with RA leads to the generation of valuable information on the individual’s balance ability [7].

Postural sway measurements are usually obtained in standing or dynamic conditions with the subjects eyes opened or closed. Posturography uses force plates to measure and software to analyse the displacement of the body’s centre of pressure (CoP). The output consists of several sway parameters describing the three-dimensional extent of postural sway, thus the individual postural control and balance mechanisms [22, 23]. In the literature it has been reported that individuals with a history of falls and RA have higher postural sway, and sway parameters can be used to differentiate between fallers and non-fallers in this population [11]. However, there are a limited number of studies in which postural sway and prospective falls in rheumatoid patients was investigated [12]. Therefore, the purpose of this study was to analyse the associations between clinical characteristics, balance performance, and postural sway parameters measured on a force platform and prospective falls in a sample of persons with RA.

## Methods

### Study design and sample

This prospective, observational study was conducted at the Centre for Muscle and Bone Research, and the Department of Rheumatology and Clinical Immunology at the Charité – Universitätsmedizin Berlin. Inclusion criteria included age ≥ 18 years, a confirmed diagnosis of rheumatoid arthritis according to the 2010 classification criteria of the American College of Rheumatology [24], the ability to walk with or without a walking aid, and written informed consent for participation in the study. Individuals were excluded if they had (1) an injury affecting muscle function in the last 3 months, (2) an acute illness or exacerbation of a chronic disease affecting muscle function, (3) an existing pregnancy, or (4) further contraindications according to §28d of the German X-ray Regulation (“Röntgenverordnung”), i.e., a dose of more than 10 millisieverts in the past ten years [10, 25]. The ethical committee of the Charité – Universitätsmedizin Berlin approved the protocol (EA4/155/16), as well as the German Radiation Protection Office (Z 5–2246/2–2016–145). The study has been registered at the German Clinical Trials Register (DRKS) and the WHO International Clinical Trials Registry Platform (ICTRP) since 16 March 2017 (DRKS00011873).

As this analysis was linked to a cross-sectional study investigating the prevalence of sarcopenia in RA (SarkoRA), the sample size calculation was based on the assumption of a prevalence of 25% in RA patients with a two-sided 95% confidence interval. A sample size of *n* = 289 was calculated based on the sample size estimation software nQuery + nTerim 3.0.

### Patient and public involvement

In order to address the patient’s perspective throughout the research process, as recommended by the European League Against Rheumatism (EULAR) [26], two patient representatives were involved in the development of the research questions and the study design, the interpretation of the results and the dissemination among their peers.

### Measurements

#### Clinical characteristics

For the baseline, clinical characteristics such as age, sex, height and weight (Seca 764), body mass index (BMI), RA disease duration, C-reactive protein (CRP) and the Disease Activity Score (DAS28_CRP_) [27] were collected.

#### Performance tests and questionnaires

Patients were evaluated with the Short Physical Performance Battery (SPPB), the FICSIT-4 including the Romberg test, the Health Assessment Questionnaire (HAQ) and the Activity-specific Balance Confidence Scale (ABC-scale) for the baseline. The SPPB score was composed of the results of a 4-m gait speed test, the chair rise test (CRT), and the Romberg test, for which the subjects received 0–4 points for each of the tests, for a maximum score of 12, in accordance with Guralnik et al. [16]. The validity and reliability of the SPPB for predicting falls has been demonstrated in several studies [17, 18, 28].

In the FICSIT-4 assessment the standing balance of the Romberg, semitandem, tandem and one-leg stances are evaluated by using a composite score. The instrument has a good reliability and validity [19]. The sum of the scores has a maximum of 5 and reflects a participants ability to hold the position for at least 10 s or not. As a continuous variable it gives a summary performance score (range, 0–5) with higher scores indicating a better standing balance. For the study, a dichotomous variable “FICSIT-4_dicho” was integrated in the analysis in order to reflect poor balance (0–4 points) and good balance (5 points, reference group).

The ABC-scale is used to evaluate the self-reported balancing confidence [29]. The respondents were required to rate their self-confidence with regard to 16 more or less challenging tasks of daily life. The value is expressed as a percentage, with 100% representing the highest possible level of confidence. The instrument can show high associations with fear of falling [30] and falls [31–33].

The assessment of disability was determined by the HAQ [34], which is used to evaluate 8 dimensions of daily life activities with items rated from 0 (no impairments) to 3 (severely disabled).

#### Posturography

For the assessment of postural sway the participants had to perform the Romberg test with a Romberg, semitandem, tandem and a left- and right-sided one-leg stance with their eyes open on the Leonardo Mechanograph® Ground Reaction Force Plate (Novotec Medical GmbH, Pforzheim, Germany, software package 4.4) in accordance with the standard procedures as recommended by the International Society of Posture and Gait Research [35]. For all measurements, a recording frequency of 800 Hz was used. The CoP data were filtered using a low-pass FIR filter with 30 sampling points and a cut-off frequency of 8 Hz. During the assessment, subjects were allowed to wear their own flat shoes and clothing.

Participants were instructed to hold the four positions of the Romberg test for 10 s as measured by a stopwatch. In the cases that a subject refused, failed or held a position for less than 10 s, the time was noted in seconds and no further position with a higher degree of difficulty was tried. The one-leg stand was explored for both legs.

 During the assessement, the Leonardo software recorded the movement of the centre of pressure (CoP), which describes the postural sway. Relevant outcome parameters were included in the analysis, such as path length (PLen), area of sway (StdElA), mean velocity of CoP (VmeanCoP), and path length and velocity in the anterior–posterior (PLenY, VmeanY) and medio-lateral (PLenX, VmeanX) directions (Table 1) [36].

**Table 1:** Abbreviations and descriptions of the CoP parameters

Abbreviation	Variable	Description
**Path-related CoP parameters**		
PLen	Path length of CoP	Total path length of the CoP during the measurement, in mm
PLenX	Medio-lateral component of the path length of CoP	Total path length of the CoP in medio-lateral direction, in mm
PLenY	Anterior–posterior component of the path length of CoP	Total path length of the CoP in anterior–posterior direction, in mm
**Area-related CoP parameters**		
StdElA	Area of sway	Standard ellipse area including 90% of all CoP points during the measurement, in cm^2^
**Speed-related CoP parameters**		
VmeanCoP	Mean velocity of CoP	Mean speed of the movement of the CoP over the time of the test path length/ duration, in cm/s
VmeanX	Mean velocity of ML	Average speed of CoP movement in medio-lateral direction, in mm/s
VmeanY	Mean velocity of AP	Average speed of CoP movement in anterior–posterior direction, in mm/s

Furthermore, the analysis contains dichotomous variables, which are related to whether the standing positions Romberg, semitandem, tandem and one-leg stand could be held for 10 s or not, e.g. “Romberg_Balance failed”.

#### Fall assessment

After the baseline assessment, all participants were asked to complete a fall diary over a period of one year. They began fall monitoring within the same month if the baseline assessment was conducted before the 15^th^ and in the following month if it was conducted after the 15^th^. Every 3 months they had to report and specify their falls in a standardised protocol. If the subject experienced a fall, further questions about the fall situation had to be answered. The subjects had to describe in their own words the cause of the fall and any conditions that might have led to the fall. If there were any uncertainties regarding the documentation, the participants were contacted via telephone by the study centre. Falls were defined, in accordance with the consensus statement of the ProFaNE group, as ‘‘an unexpected event in which the participants come to rest on the ground, floor, or lower level’’ [37]. Falls due to syncope or accidents were excluded. Patients were divided into a falls group (one or more falls) and a non-faller group (no fall), in accordance with their reports during the one-year follow-up.

### Statistical analysis

The descriptive data are presented as mean and standard deviation (SD) for continuous and normally distributed variables, and median and interquartile ranges for skewed variables. Categorical variables are reported in frequencies and percentages. The group of non-fallers and fallers were compared with t-tests for independent samples, Mann–Whitney-U-tests or Chi^2^ tests.

Postural sway variables were stratified into quartiles in order to integrate the missing data of failed balance assessments. Quartile 1 (Q1) included the subjects with the best results and was defined as the reference. Quartile 4 (Q4) contained the persons with the worst results and those who failed the assessment.

In the first analysis step, univariate logistic regression was performed followed by a multiple logistic regression with an adjustment for age and sex in order to explore associations between variables of balance and performance with regard to falling. The results for this are presented along with the odds ratio (OR), the 95% confidence interval (CI) and the p-value (2-sided). For the CoP variables, the logistic regression model was used to estimate the OR for falling in each quartile (Q2-Q4) compared to the best quartile (Q1).

Receiver-operating characteristics (ROC) were analysed to determine the quality of the prediction and the area under the curve (AUC) along with the associated 95% CI and p-value also reported. The ROC analyses did not provide specific cut-off values; therefore, these results are not shown.

## Results

### Characteristics of baseline assessment

Two hundred thirty-eight subjects between 24 and 85 years of age completed the one-year follow-up (82.3%). The mean age was 60.2 ± 11.6 years. The majority of the sample consisted of females (*n* = 187, 78.6%). There were 48 (20.2%) persons who reported a fall episode in the observation period, of which 83.3% were female. In Table 2 and 3 the clinical and performance characteristics are presented of the responder sample for the baseline. A flowchart of the study and a responder-analysis are reported elsewhere [10].

**Table 2:** Clinical characteristics of the responder sample at baseline

Variables	Responder *n* = 238				
	**Non-Fallers**		**Fallers**		
	**n**	**mean (± SD)**	**n**	**mean (± SD)**	***p*-value**^**1**^
**Clinical characteristics**					
Age	190	59.2 (± 11.9)	48	63.7 (± 9.5)	**0.016**
Females	147	58.4 (± 11.9)	40	63.6 (± 9.9)	**0.013**
Females, n, (%)	190	147 (77.4)	48	40 (83.3)	0.368
Height (m)	190	1.67 (± 0.08)	48	1.64 (± 0.07)	**0.017**
Weight (kg)	190	77.3 (± 14.0)	48	73.1 (± 12.0)	0.059
BMI (kg/m^2^)	190	27.1 (± 4.5)	48	26.5 (± 4.0)	0.401
RA disease duration (y)^a^	190	9.0 (4.0–16.0)	48	11.0 (5.0–19.7)	0.33
DAS28_CRP_ (score)^a^	189	2.07 (1.62–2.85)	45	2.33 (1.69–3.25)	0.161
Low disease activity ≤ 3.2, n, (%)		154 (81.1)		33 (66.8)	0.229
Moderate disease activity 3.2 ≤ 5.1, n, (%)		34 (17.9)		12 (25.0)	
High disease activity > 5.1, n, (%)		1 (0.5)		0	

^1^*p*-value of unpaired t-test or Mann–Whitney-U-Test or Chi^2^-test, bold values significant difference between fallers and non-fallers

^a^Data are presented as median (interquartile range)

**Table 3:** Balance and performance characteristics of the baseline assessment of the responder sample

Variables	Responder *n* = 238				
	**Non-Fallers**		**Fallers**		
	**n**	**mean (± SD)**	**n**	**mean (± SD)**	***p*-value**^**1**^
**Balance and performance assessments**					
HAQ (score)^a^	190	0.37 (0.0–1.12)	48	0.81 (0.41–1.5)	**0.001**
ABC-scale (%)^a^	190	91.12 (75.47–97.31)	48	88.75 (76.56–95.70)	0.32
SPPB (score)^a^	190	11.0 (11.0–12.0)	48	11.0 (10.0–12.0)	0.645
FICSIT-4 (score)^a^	190	5.0 (0.0)	48	5.0 (4.0–5.0)	**0.030**
FICSIT-4_dicho (0–4), n, (%)	190	28 (14.7)	48	14 (29.2)	**0.019**
Romberg_Balance failed, n, (%)	190	0	48	0	
Semitandem_Balance failed, n, (%)	190	0	48	0	
Tandem_Balance failed, n, (%)	190	18 (9.5)	48	5 (10.4)	0.843
One leg left_Balance failed, n, (%)	190	36 (18.9)	48	16 (33.3)	**0.031**
One leg right_Balance failed, n, (%)	190	54 (28.4)	48	20 (41.7)	0.076

^1^*p*-value of unpaired t-test or Mann–Whitney-U-Test or Chi^2^-test, bold values significant difference between fallers and non-fallers

^a^Data are presented as median (interquartile range)

Patients who experienced falls were significantly older (*p* = 0.016) and more limited in their activities of daily life (HAQ score, *p* = 0.001) (Tables 2; 3). Balance assessment with the FICSIT-4, revealed that patients with falls had a significant greater range in the FICSIT-4 score (IQR 4.0–5.0; *p* = 0.03) and a significantly higher proportion of fallers (29.2%) were poor performers, scoring 0–4 in FICSIT-4 (FICSIT-4_dicho, *p* = 0.019). The failure rate of performing one-leg stances was significantly higher in the falls group (p_left_ = 0.031). Fallers demonstrated lower balance confidence (ABC-scale), but this was not significant (*p* = 0.321) (Table 3).

In Table 4 the quartiles are shown along with the number of subjects and percentages by follow-up fall status for each test position and sway parameter of the balance assessment on the Leonardo Mechanograph®. In general, the percentages of subjects who failed the assessment or achieved the worst results (Q4) were higher in the falls group than in the non-faller group. Significant differences between fallers and non-fallers could be found in the sway parameters of the Romberg, semitandem and tandem stance. For Romberg, the path length and the velocity of CoP both in the medio-lateral direction showed a significant difference between the two groups (*p* = 0.039). For the semitandem stance, the path length and the velocity of CoP in the medio-lateral direction (*p* = 0.019 and 0.020, respectively), the area of sway (*p* = 0.029), the path length in total and the speed of the movement of the CoP over time (*p* = 0.039) were also significantly different between the groups. For the tandem stance, the path length in total and the speed of the movement of the CoP over time (both *p* = 0.034) as well as the path length and the velocity of CoP in the anterior–posterior direction (both *p* = 0.015) showed once again significant differences between the groups (Table 4).

**Table 4:** Baseline balance assessment on the Leonardo Mechanograph® for follow-up fallers and non-fallers, *n* = 238

				**Non-Fallers**		**Fallers**		
**Test position**	**Variables**	**Quartile range**		**n**	**%**	**n**	**%**	*p*-value*
Romberg	PLen [in mm]	≤ 134.81	Q1 (best)	50	26.3	10	20.8	0.364
		134.82—168.14	Q2 (good)	46	24.2	13	27.1	
		168.15—212.81	Q3 (fair)	49	25.8	10	20.8	
		≥ 212.82	Q4 (poor, failed)	44	23.2	15	31.3	
	PLenX [in mm]	≤ 84.02	Q1 (best)	52	27.4	8	16.7	**0.039**
		84.03—108.87	Q2 (good)	48	25.3	11	22.9	
		108.29—139.72	Q3 (fair)	47	24.7	12	25.0	
		≥ 139.73	Q4 (poor, failed)	42	22.1	17	35.4	
	PLenY [in mm]	≤ 78.58	Q1 (best)	46	24.2	14	29.2	0.853
		78.59—101.10	Q2 (good)	49	25.8	10	20.8	
		101.11—130.33	Q3 (fair)	50	26.3	9	18.8	
		≥ 130.34	Q4 (poor, failed)	44	23.2	15	31.3	
	StdElA [in cm^2^]	≤ 1.07	Q1 (best)	46	24.2	14	29.2	0.637
		1.08—1.69	Q2 (good)	49	25.8	10	20.8	
		1.70—2.61	Q3 (fair)	52	27.4	7	14.6	
		≥ 2.62	Q4 (poor, failed)	42	22.1	17	35.4	
	VmeanCoP [in cm/s]	≤ 1.35	Q1 (best)	50	26.3	10	20.8	0.364
		1.36—1.68	Q2 (good)	46	24.2	13	27.1	
		1.69—2.13	Q3 (fair)	49	25.8	10	20.8	
		≥ 2.14	Q4 (poor, failed)	44	23.2	15	31.3	
	VmeanX [in mm/s]	≤ 8.40	Q1 (best)	52	27.4	8	16.7	**0.039**
		8.41—10.89	Q2 (good)	48	25.3	11	22.9	
		10.90—13.97	Q3 (fair)	47	24.7	12	25.0	
		≥ 13.98	Q4 (poor, failed)	42	22.1	17	35.4	
	VmeanY [in mm/s]	≤ 7.86	Q1 (best)	46	24.2	14	29.2	0.853
		7.87—10.11	Q2 (good)	49	25.8	10	20.8	
		10.12—13.03	Q3 (fair)	50	26.3	9	18.8	
		≥ 13.04	Q4 (poor, failed)	44	23.2	15	31.3	
Semitandem	PLen [in mm]	≤ 161,93	Q1 (best)	54	28.4	6	12.5	**0.039**
		161.94—213.16	Q2 (good)	46	24.2	13	27.1	
		213.17—270.09	Q3 (fair)	45	23.7	14	29.2	
		≥ 270.10	Q4 (poor, failed)	44	23.2	15	31.3	
	PLenX [in mm]	≤ 108.11	Q1 (best)	53	27.9	7	14.6	**0.019**
		108.12—141.45	Q2 (good)	50	26.3	9	18.8	
		141.46—184.19	Q3 (fair)	42	22.1	17	35.4	
		≥ 184.2	Q4 (poor, failed)	44	23.2	15	31.3	
	PLenY [in mm]	≤ 99.88	Q1 (best)	51	26.8	9	18.8	0.138
		99.89—123.97	Q2 (good)	46	24.2	13	27.1	
		123.98—175.59	Q3 (fair)	50	26.3	9	18.8	
		≥ 175.6	Q4 (poor, failed)	42	22.1	17	35.4	
	StdElA [in cm^2^]	≤ 1.31	Q1 (best)	51	26.8	8	16.7	**0.029**
		1.32—1.93	Q2 (good)	49	25.8	11	22.9	
		1.94—2.90	Q3 (fair)	48	25.3	11	22.9	
		≥ 2.91	Q4 (poor, failed)	41	21.6	18	37.5	
	VmeanCoP [in cm/s]	≤ 1.62	Q1 (best)	54	28.4	6	12.5	**0.039**
		1.63—2.13	Q2 (good)	46	24.2	13	27.1	
		2.14—2.70	Q3 (fair)	45	23.7	14	29.2	
		≥ 2.71	Q4 (poor, failed)	44	23.2	15	31.3	
	VmeanX [in mm/s]	≤ 10.81	Q1 (best)	52	27.4	7	14.6	**0.020**
		10.82—14.15	Q2 (good)	51	26.8	9	18.8	
		14.16—18.42	Q3 (fair)	42	22.1	17	35.4	
		≥ 18.43	Q4 (poor, failed)	44	23.2	15	31.3	
	VmeanY [in mm/s]	≤ 9.99	Q1 (best)	50	26.3	9	18.8	0.145
		10.00—12.40	Q2 (good)	47	24.7	13	27.1	
		12.41—17.56	Q3 (fair)	50	26.3	9	18.8	
		≥ 17.57	Q4 (poor, failed)	42	22.1	17	35.4	
Tandem	PLen [in mm]	≤ 330.31	Q1 (best)	48	25.3	6	12.5	**0.034**
		330.32—422.31	Q2 (good)	43	22.6	10	20.8	
		422.32—583.23	Q3 (fair)	42	22.1	12	25.0	
		≥ 583.24	Q4 (poor, failed)	56	29.5	20	41.7	
	PLenX [in mm]	≤ 227.95	Q1 (best)	47	24.7	7	14.6	0.095
		227.96—293.50	Q2 (good)	44	23.2	9	18.8	
		293.51—391.22	Q3 (fair)	40	21.1	14	29.2	
		≥ 391.23	Q4 (poor, failed)	58	30.5	18	37.5	
	PLenY [in mm]	≤ 187.51	Q1 (best)	46	24.2	8	16.7	**0.015**
		187.52—256.19	Q2 (good)	47	24.7	6	12.5	
		256.20—363.06	Q3 (fair)	42	22.1	12	25.0	
		≥ 363.07	Q4 (poor, failed)	54	28.4	22	45.8	
	StdElA [in cm^2^]	≤ 1.95	Q1 (best)	46	24.2	8	16.7	0.252
		1.96—3.13	Q2 (good)	42	22.1	11	22.9	
		3.14—4.73	Q3 (fair)	43	22.6	11	22.9	
		≥ 4.74	Q4 (poor, failed)	58	30.5	18	37.5	
	VmeanCoP [in cm/s]	≤ 3.30	Q1 (best)	48	25.3	6	12.5	**0.034**
		3.31—4.22	Q2 (good)	43	22.6	10	20.8	
		4.23—5.83	Q3 (fair)	42	22.1	12	25.0	
		≥ 5.84	Q4 (poor, failed)	56	29.5	20	41.7	
	VmeanX [in mm/s]	≤ 22.80	Q1 (best)	47	24.7	7	14.6	0.095
		22.81—29.35	Q2 (good)	44	23.2	9	18.8	
		29.36—39.12	Q3 (fair)	40	21.1	14	29.2	
		≥ 39.13	Q4 (poor, failed)	58	30.5	18	37.5	
	VmeanY [in mm/s]	≤ 18.75	Q1 (best)	46	24.2	8	16.7	**0.015**
		18.76—25.62	Q2 (good)	47	24.7	6	12.5	
		25.63—36.31	Q3 (fair)	42	22.1	12	25.0	
		≥ 36.32	Q4 (poor, failed)	54	28.4	22	45.8	
One leg left	PLen [in mm]	≤ 358.95	Q1 (best)	37	19.5	10	20.8	0.127
		358.96—513.28	Q2 (good)	43	22.6	3	6.3	
		513.29—649.39	Q3 (fair)	36	18.9	10	20.8	
		≥ 649.4	Q4 (poor, failed)	73	38.4	25	52.1	
	PLenX [in mm]	≤ 254.59	Q1 (best)	38	20.0	9	18.8	0.088
		254.60—371.00	Q2 (good)	41	21.6	5	10.4	
		371.01—465.43	Q3 (fair)	38	20.0	8	16.7	
		≥ 465.44	Q4 (poor, failed)	72	37.9	26	54.2	
	PLenY [in mm]	≤ 205.57	Q1 (best)	37	19.5	10	20.8	0.139
		205.58—279.09	Q2 (good)	41	21.6	5	10.4	
		279.10—365.03	Q3 (fair)	39	20.5	7	14.6	
		≥ 365.04	Q4 (poor, failed)	72	37.9	26	54.2	
	StdElA [in cm^2^]	≤ 2.65	Q1 (best)	39	20.5	8	16.7	0.216
		2.66—3.84	Q2 (good)	38	20.0	8	16.7	
		3.85—5.69	Q3 (fair)	38	20.0	8	16.7	
		≥ 5.7	Q4 (poor, failed)	74	38.9	24	50.0	
	VmeanCoP [in cm/s]	≤ 3.59	Q1 (best)	37	19.5	10	20.8	0.127
		3.60—5.13	Q2 (good)	43	22.6	3	6.3	
		5.14—6.49	Q3 (fair)	36	18.9	10	20.8	
		≥ 6.5	Q4 (poor, failed)	73	38.4	25	52.1	
	VmeanX [in mm/s]	≤ 25.46	Q1 (best)	38	20.0	9	18.8	0.088
		25.47—37.10	Q2 (good)	41	21.6	5	10.4	
		37.11—46.54	Q3 (fair)	38	20.0	8	16.7	
		≥ 46.55	Q4 (poor, failed)	72	37.9	26	54.2	
	VmeanY [in mm/s]	≤ 20.56	Q1 (best)	37	19.5	10	20.8	0.139
		20.57—27.91	Q2 (good)	41	21.6	5	10.4	
		27.92—36.50	Q3 (fair)	39	20.5	7	14.6	
		≥ 36.51	Q4 (poor, failed)	72	37.9	26	54.2	
One leg right	PLen [in mm]	≤ 379.57	Q1 (best)	35	18.4	6	12.5	0.142
		379.58—489.95	Q2 (good)	33	17.4	8	16.7	
		489.96—651.19	Q3 (fair)	35	18.4	6	12.5	
		≥ 651.2	Q4 (poor, failed)	86	45.3	28	58.3	
	PLenX [in mm]	≤ 264.69	Q1 (best)	36	18.9	5	10.4	0.094
		264.70—358.00	Q2 (good)	33	17.4	8	16.7	
		358.01—466.27	Q3 (fair)	34	17.9	7	14.6	
		≥ 466.28	Q4 (poor, failed)	86	45.3	28	58.3	
	PLenY [in mm]	≤ 214.87	Q1 (best)	33	17.4	8	16.7	0.336
		214.88—268.04	Q2 (good)	34	17.9	7	14.6	
		268.05—371.17	Q3 (fair)	35	18.4	6	12.5	
		≥ 371.18	Q4 (poor, failed)	87	45.8	27	56.3	
	StdElA [in cm^2^]	≤ 2.59	Q1 (best)	32	16.8	9	18.8	0.145
		2.60—3.79	Q2 (good)	37	19.5	4	8.3	
		3.80—6.19	Q3 (fair)	35	18.4	6	12.5	
		≥ 6.2	Q4 (poor, failed)	85	44.7	29	60.4	
	VmeanCoP [in cm/s]	≤ 3.80	Q1 (best)	35	18.4	6	12.5	0.142
		3.81—4.90	Q2 (good)	33	17.4	8	16.7	
		4.91—6.51	Q3 (fair)	35	18.4	6	12.5	
		≥ 6.52	Q4 (poor, failed)	86	45.3	28	58.3	
	VmeanX [in mm/s]	≤ 26.47	Q1 (best)	36	18.9	5	10.4	0.094
		26.48—35.80	Q2 (good)	33	17.4	8	16.7	
		35.81—46.63	Q3 (fair)	34	17.9	7	14.6	
		≥ 46.64	Q4 (poor, failed)	86	45.3	28	58.3	
	VmeanY [in mm/s]	≤ 21.49	Q1 (best)	33	17.4	8	16.7	0.336
		21.50—26.80	Q2 (good)	34	17.9	7	14.6	
		26.81—37.12	Q3 (fair)	35	18.4	6	12.5	
		≥ 37.13	Q4 (poor, failed)	87	45.8	27	56.3	

^*^p-values of Mann–Whitney-U-Test: bold values sign. p-value with p < 0.05

### Factors associated with prospective falls

Regarding clinical characteristics, a significant association with prospective falls was found for age (OR = 1.04; CI 1.01–1.07, *p* = 0.017). There were no associations with fall risk found for sex, RA disease duration, and disease activity (Table 5).

**Table 5:** Unadjusted and adjusted associations for fall risk

		**Unadjusted**						**Adjusted for age and sex**^**a**^					
**Predictors**		**OR**	**[95% CI]**	***p*-value**	**AUC**	**[95% CI]**	***p*-value**	**OR**	**[95% CI]**	*p*-value	**AUC**	**[95% CI]**	***p*-value**
**Clinical characteristics**													
Age		**1.04**	[1.01–1.07]	**0.017**	0.60	[0.52–0.69]	0.026	n.a					
Sex^a^		1.46	[0.64–3.36]	0.370	0.47	[0.38–0.56]	0.523	n.a					
DAS28_CRP_		1.28	[0.93–1.76]	0.125	0.57	[0.47–0.66]	0.161	1.35	[0.97–1.87]	0.072	0.64	[0.55–0.72]	0.004
RA disease duration		1.02	[0.98–1.05]	0.281	0.55	[0.45–0.64]	0.331	1.01	[0.97–1.04]	0.769	0.61	[0.53–0.7	0.014
**Balance and performance assessments**													
HAQ		**1.62**	[1.1–2.38]	**0.014**	0.65	[0.57–0.73]	0.002	1.52	[1.01–2.27]	**0.043**	0.65	[0.56–0.74]	0.001
ABC-scale		1.00	[0.98–1.01]	0.971	0.45	[0.36–0.54]	0.322	1.00	[0.99–1.02]	0.566	0.62	[0.53–0.7]	0.013
SPPB		0.97	[0.80–1.17]	0.736	0.48	[0.39–0.57]	0.666	1.10	[0.89–1.35]	0.394	0.62	[0.53–0.7]	0.014
FICSIT-4 (score)		0.74	[0.50–1.1]	0.137	0.43	[0.34–0.53]	0.150	0.94	[0.60–1.46]	0.768	0.61	[0.53–0.70]	0.015
FICSIT-4_dicho	Poor balance (score 0–4)	**2.38**	[1.13–5.0]	**0.022**	0.43	[0.33–0.52]	0.123	1.65	[0.72–3.8]	0.239	0.62	[0.54–0.71]	0.008
	Good balance (score 5)	Reference						Reference					
One leg left_Balance	Failed	**2.14**	[1.06–4.31]	**0.034**	0.43	[0.33–0.52]	0.124	1.52	[0.69–3.34]	0.297	0.62	[0.53–0.71]	0.010
	Done	Reference						Reference					
One leg right_Balance	Failed	1.80	[0.93–3.46]	0.079	0.43	[0.34–0.53]	0.156	1.27	[0.6–2.68]	0.531	0.62	[0.53–0.70]	0.012
	Done	Reference						Reference					

^a^Male = Reference

In terms of balance and performance assessments, the univariate logistic regression analysis revealed significant associations for HAQ score (OR = 1.62; CI 1.1–2.38, *p* = 0.014), low FICSIT-4 score (0–4) (OR = 2.38; CI 1.13–5.0, *p* = 0.022) and failed one-leg stand (OR_left_ = 2.14; CI 1.06–4.31, *p* = 0.034). The ABC-scale and SPPB remained non-significant.

The multiple logistic regression for FICSIT-4_dicho showed a higher and significant AUC (0.62; CI 0.54–0.71, *p* = 0.008), thus a better predictive quality for falls than the univariate regression. Similar results were obtained for the performance of one-leg stand. The probability of falling increased significantly in the case that the subject had failed the test. The AUC of the adjusted association was higher at 0.62 (CI 0.53–0.71, *p* = 0.010) and significant compared to the unadjusted association (AUC = 0.43; CI 0.33–0.52, *p* = 0.124) (Table 5).

In Table 6 the results are shown of the univariate and multiple logistic regression analysis of the postural sway parameters when using falls as a dependent variable. In general, subjects within the worst quartile (Q4) had increased odds of falls compared to subjects of the best quartile (Q1) and in most cases also higher odds than those of the other quartiles (Q2 and Q3). For the univariate logistic regression analysis, the highest and significant OR in Q4 could be found for Romberg PLenX and VmeanX (OR = 2.63; CI 1.03–6,69, *p* = 0.042), semitandem PLen, VmeanCoP (OR = 3.07; CI 1.10–8.57, *p* = 0.032) and StdElA (OR = 2.80; CI 1.11–7.08, *p* = 0.030) and tandem PLen and VmeanCoP (OR = 2.86; CI 1.06–7.69, *p* = 0.038). The AUCs of those unadjusted and significant models were between 0.59 and 0.61 (Table 6).

**Table 6:** Unadjusted and adjusted associations between balance sway parameters and fall risk

			Unadjusted								Adjusted for age and sex^**a**^							
Test position	Predictors	Quartiles	OR	95% CI		*p*-value	AUC	95% CI		***p***-value	OR	95% CI		*p*-value	AUC	95% CI		***p***-value
Romberg	PLen	Q1 (best)	Reference				0.54	0.45	0.63	0.379	Reference				0.62	0.54	0.71	0.009
		Q2 (good)	1.41	0.57	3.53	0.766					1.21	0.47	3.11	0.699				
		Q3 (fair)	1.02	0.39	2.67	0.660					0.89	0.32	2.45	0.822				
		Q4 (poor, failed)	1.70	0.69	4.18	0.915					1.15	0.42	3.15	0.792				
	PLenX	Q1 (best)	Reference				0.59	0.50	0.68	**0.046**	Reference				0.64	0.56	0.73	0.002
		Q2 (good)	1.49	0.55	4.02	0.431					1.31	0.48	3.60	0.599				
		Q3 (fair)	1.66	0.62	4.41	0.310					1.56	0.57	4.27	0.384				
		Q4 (poor, failed)	2.63	1.03	6.69	0.042					2.12	0.78	5.72	0.139				
	PLenY	Q1 (best)	Reference				0.51	0.41	0.61	0.858	Reference				0.63	0.54	0.72	0.005
		Q2 (good)	0.67	0.27	1.66	0.387					0.63	0.25	1.60	0.332				
		Q3 (fair)	0.59	0.23	1.50	0.267					0.48	0.18	1.31	0.150				
		Q4 (poor, failed)	1.12	0.48	2.59	0.791					0.74	0.29	1.89	0.533				
	StdElA	Q1 (best)	Reference				0.52	0.42	0.62	0.647	Reference				0.67	0.59	0.75	0.000
		Q2 (good)	0.67	0.27	1.66	0.387					0.57	0.22	1.44	0.228				
		Q3 (fair)	0.44	0.16	1.19	0.106					0.34	0.12	0.97	0.042				
		Q4 (poor, failed)	1.33	0.58	3.03	0.496					0.97	0.40	2.36	0.943				
	VmeanCoP	Q1 (best)	Reference				0.54	0.45	0.63	0.379	Reference				0.62	0.54	0.71	0.009
		Q2 (good)	1.41	0.57	3.53	0.460					1.21	0.47	3.11	0.699				
		Q3 (fair)	1.02	0.39	2.67	0.967					0.89	0.32	2.45	0.822				
		Q4 (poor, failed)	1.70	0.70	4.18	0.244					1.15	0.42	3.15	0.792				
	VmeanX	Q1 (best)	Reference				0.59	0.50	0.68	**0.046**	Reference				0.64	0.56	0.73	0.002
		Q2 (good)	1.49	0.55	4.02	0.431					1.31	0.48	3.60	0.599				
		Q3 (fair)	1.66	0.62	4.41	0.310					1.56	0.57	4.27	0.384				
		Q4 (poor, failed)	2.63	1.03	6.69	0.042					2.12	0.78	5.72	0.139				
	VmeanY	Q1 (best)	Reference				0.51	0.41	0.61	0.858	Reference				0.63	0.54	0.72	0.005
		Q2 (good)	0.67	0.27	1.66	0.387					0.63	0.25	1.60	0.332				
		Q3 (fair)	0.59	0.23	1.50	0.267					0.48	0.18	1.31	0.150				
		Q4 (poor, failed)	1.12	0.48	2.59	0.791					0.74	0.29	1.89	0.533				
Semitandem	PLen	Q1 (best)	Reference				0.59	0.51	0.68	**0.046**	Reference				0.65	0.56	0.73	0.002
		Q2 (good)	2.54	0.90	7.23	0.080					2.11	0.71	6.32	0.182				
		Q3 (fair)	2.80	0.99	7.88	0.051					2.21	0.73	6.70	0.161				
		Q4 (poor, failed)	3.07	1.10	8.57	0.032					2.11	0.61	7.34	0.240				
	PLenX	Q1 (best)	Reference				0.61	0.52	0.69	**0.023**	Reference				0.66	0.57	0.74	0.001
		Q2 (good)	1.36	0.47	3.94	0.567					1.11	0.37	3.31	0.856				
		Q3 (fair)	3.06	1.16	8.08	0.023					2.49	0.89	6.97	0.082				
		Q4 (poor, failed)	2.58	0.97	6.89	0.058					1.74	0.55	5.55	0.349				
	PLenY	Q1 (best)	Reference				0.57	0.47	0.66	0.151	Reference				0.64	0.56	0.72	0.002
		Q2 (good)	1.60	0.63	4.09	0.325					1.40	0.53	3.71	0.493				
		Q3 (fair)	1.02	0.37	2.78	0.969					0.76	0.26	2.24	0.621				
		Q4 (poor, failed)	2.29	0.93	5.67	0.072					1.43	0.48	4.29	0.525				
	StdElA	Q1 (best)	Reference				0.60	0.51	0.69	**0.035**	Reference				0.65	0.56	0.73	0.002
		Q2 (good)	1.43	0.53	3.86	0.479					1.23	0.44	3.41	0.694				
		Q3 (fair)	1.46	0.54	3.94	0.454					1.23	0.44	3.44	0.693				
		Q4 (poor, failed)	2.80	1.11	7.08	0.030					2.08	0.76	5.70	0.156				
	VmeanCoP	Q1 (best)	Reference				0.59	0.51	0.68	**0.046**	Reference				0.65	0.56	0.73	0.002
		Q2 (good)	2.54	0.90	7.23	0.080					2.11	0.71	6.32	0.182				
		Q3 (fair)	2.80	0.99	7.88	0.051					2.21	0.73	6.70	0.161				
		Q4 (poor, failed)	3.07	1.10	8.57	0.032					2.11	0.61	7.34	0.240				
	VmeanX	Q1 (best)	Reference				0.61	0.52	0.69	**0.024**	Reference				0.66	0.57	0.74	0.001
		Q2 (good)	1.31	0.45	3.79	0.617					1.04	0.34	3.13	0.947				
		Q3 (fair)	3.01	1.14	7.93	0.026					2.40	0.85	6.76	0.097				
		Q4 (poor, failed)	2.53	0.95	6.77	0.064					1.67	0.52	5.39	0.389				
	VmeanY	Q1 (best)	Reference				0.57	0.47	0.66	0.158	Reference				0.64	0.56	0.72	0.003
		Q2 (good)	1.54	0.60	3.93	0.370					1.34	0.51	3.55	0.550				
		Q3 (fair)	1.00	0.37	2.73	1.000					0.74	0.25	2.19	0.589				
		Q4 (poor, failed)	2.25	0.91	5.56	0.080					1.39	0.46	4.18	0.558				
Tandem	PLen	Q1 (best)	Reference				0.60	0.51	0.68	**0.041**	Reference				0.63	0.55	0.72	0.005
		Q2 (good)	1.86	0.62	5.55	0.265					1.46	0.46	4.62	0.524				
		Q3 (fair)	2.29	0.79	6.62	0.128					1.63	0.51	5.20	0.411				
		Q4 (poor, failed)	2.86	1.06	7.69	0.038					1.81	0.55	5.92	0.326				
	PLenX	Q1 (best)	Reference				0.58	0.49	0.66	0.107	Reference				0.62	0.54	0.71	0.008
		Q2 (good)	1.37	0.47	4.00	0.561					1.03	0.33	3.18	0.961				
		Q3 (fair)	2.35	0.86	6.39	0.094					1.47	0.49	4.41	0.487				
		Q4 (poor, failed)	2.08	0.80	5.41	0.131					1.19	0.38	3.73	0.759				
	PLenY	Q1 (best)	Reference				0.61	0.52	0.70	**0.019**	Reference				0.64	0.56	0.72	0.002
		Q2 (good)	0.73	0.24	2.28	0.593					0.66	0.21	2.10	0.480				
		Q3 (fair)	1.64	0.61	4.41	0.324					1.38	0.48	3.96	0.547				
		Q4 (poor, failed)	2.34	0.95	5.76	0.064					1.62	0.57	4.62	0.364				
	StdElA	Q1 (best)	Reference				0.55	0.46	0.64	0.268	Reference				0.62	0.53	0.71	0.011
		Q2 (good)	1.51	0.55	4.10	0.423					1.42	0.51	3.94	0.504				
		Q3 (fair)	1.47	0.54	4.00	0.450					1.15	0.41	3.27	0.791				
		Q4 (poor, failed)	1.78	0.71	4.47	0.216					1.08	0.38	3.06	0.882				
	VmeanCoP	Q1 (best)	Reference				0.60	0.51	0.68	**0.041**	Reference				0.63	0.55	0.72	0.005
		Q2 (good)	1.86	0.62	5.55	0.265					1.46	0.46	4.62	0.524				
		Q3 (fair)	2.29	0.79	6.62	0.128					1.63	0.51	5.20	0.411				
		Q4 (poor, failed)	2.86	1.06	7.69	0.038					1.81	0.55	5.92	0.326				
	VmeanX	Q1 (best)	Reference				0.58	0.49	0.66	0.107	Reference				0.62	0.54	0.71	0.008
		Q2 (good)	1.37	0.47	4.00	0.561					1.03	0.33	3.18	0.961				
		Q3 (fair)	2.35	0.86	6.39	0.094					1.47	0.49	4.41	0.487				
		Q4 (poor, failed)	2.08	0.80	5.41	0.131					1.19	0.38	3.73	0.759				
	VmeanY	Q1 (best)	Reference				0.61	0.52	0.70	**0.019**	Reference				0.64	0.56	0.72	0.002
		Q2 (good)	0.73	0.24	2.28	0.593					0.66	0.21	2.10	0.480				
		Q3 (fair)	1.64	0.61	4.41	0.324					1.38	0.48	3.96	0.547				
		Q4 (poor, failed)	2.34	0.95	5.76	0.064					1.62	0.57	4.62	0.364				
One leg left	PLen	Q1 (best)	Reference				0.57	0.47	0.66	0.146	Reference				0.66	0.58	0.74	0.000
		Q2 (good)	0.26	0.07	1.01	0.051					0.17	0.04	0.69	0.013				
		Q3 (fair)	1.03	0.38	2.76	0.957					0.59	0.19	1.80	0.349				
		Q4 (poor, failed)	1.27	0.55	2.92	0.578					0.59	0.19	1.78	0.346				
	PLenX	Q1 (best)	Reference				0.58	0.48	0.67	0.104	Reference				0.65	0.57	0.73	0.001
		Q2 (good)	0.51	0.16	1.67	0.270					0.33	0.09	1.15	0.081				
		Q3 (fair)	0.89	0.31	2.55	0.826					0.52	0.16	1.70	0.280				
		Q4 (poor, failed)	1.52	0.65	3.58	0.333					0.71	0.23	2.20	0.554				
	PLenY	Q1 (best)	Reference				0.57	0.47	0.66	0.159	Reference				0.66	0.58	0.73	0.001
		Q2 (good)	0.45	0.14	1.44	0.179					0.34	0.10	1.15	0.084				
		Q3 (fair)	0.66	0.23	1.93	0.451					0.44	0.14	1.40	0.164				
		Q4 (poor, failed)	1.34	0.58	3.06	0.494					0.75	0.26	2.13	0.584				
	StdElA	Q1 (best)	Reference				0.55	0.46	0.65	0.238	Reference				0.62	0.53	0.70	0.013
		Q2 (good)	1.03	0.35	3.01	0.962					0.80	0.26	2.47	0.699				
		Q3 (fair)	1.03	0.35	3.01	0.962					0.80	0.25	2.53	0.708				
		Q4 (poor, failed)	1.58	0.65	3.85	0.313					0.95	0.34	2.71	0.929				
	VmeanCoP	Q1 (best)	Reference				0.57	0.47	0.66	0.146	Reference				0.66	0.58	0.74	0.000
		Q2 (good)	0.26	0.07	1.01	0.051					0.17	0.04	0.69	0.013				
		Q3 (fair)	1.03	0.38	2.76	0.957					0.59	0.19	1.80	0.349				
		Q4 (poor, failed)	1.27	0.55	2.92	0.578					0.59	0.19	1.78	0.346				
	VmeanX	Q1 (best)	Reference				0.58	0.48	0.67	0.104	Reference				0.65	0.57	0.73	0.001
		Q2 (good)	0.51	0.16	1.67	0.270					0.33	0.09	1.15	0.081				
		Q3 (fair)	0.89	0.31	2.55	0.826					0.52	0.16	1.70	0.280				
		Q4 (poor, failed)	1.52	0.65	3.58	0.333					0.71	0.23	2.20	0.554				
	VmeanY	Q1 (best)	Reference				0.57	0.47	0.66	0.159	Reference				0.66	0.58	0.73	0.001
		Q2 (good)	0.45	0.14	1.44	0.179					0.34	0.10	1.15	0.084				
		Q3 (fair)	0.66	0.23	1.93	0.451					0.44	0.14	1.40	0.164				
		Q4 (poor, failed)	1.34	0.58	3.06	0.494					0.75	0.26	2.13	0.584				
One leg right	PLen	Q1 (best)	Reference				0.56	0.47	0.65	0.170	Reference				0.62	0.54	0.71	0.008
		Q2 (good)	1.41	0.44	4.51	0.558					1.28	0.39	4.20	0.684				
		Q3 (fair)	1.00	0.29	3.40	1.000					0.77	0.21	2.86	0.699				
		Q4 (poor, failed)	1.90	0.72	4.99	0.193					1.10	0.35	3.50	0.866				
	PLenX	Q1 (best)	Reference				0.57	0.48	0.66	0.117	Reference				0.62	0.53	0.71	0.009
		Q2 (good)	1.75	0.52	5.87	0.368					1.44	0.41	5.07	0.567				
		Q3 (fair)	1.48	0.43	5.12	0.534					1.11	0.29	4.21	0.877				
		Q4 (poor, failed)	2.34	0.84	6.55	0.104					1.39	0.41	4.76	0.602				
	PLenY	Q1 (best)	Reference				0.54	0.45	0.63	0.368	Reference				0.62	0.53	0.70	0.013
		Q2 (good)	0.85	0.28	2.61	0.775					0.80	0.25	2.50	0.695				
		Q3 (fair)	0.71	0.22	2.26	0.558					0.60	0.18	2.02	0.411				
		Q4 (poor, failed)	1.28	0.53	3.10	0.584					0.75	0.26	2.18	0.598				
	StdElA	Q1 (best)	Reference				0.56	0.47	0.66	0.173	Reference				0.64	0.56	0.73	0.002
		Q2 (good)	0.38	0.11	1.37	0.140					0.38	0.11	1.38	0.142				
		Q3 (fair)	0.61	0.20	1.90	0.394					0.55	0.17	1.75	0.312				
		Q4 (poor, failed)	1.21	0.52	2.84	0.656					0.85	0.32	2.28	0.748				
	VmeanCoP	Q1 (best)	Reference				0.56	0.47	0.65	0.170	Reference				0.62	0.54	0.71	0.008
		Q2 (good)	1.41	0.44	4.51	0.558					1.28	0.39	4.20	0.684				
		Q3 (fair)	1.00	0.29	3.40	1.000					0.77	0.21	2.86	0.699				
		Q4 (poor, failed)	1.90	0.72	4.99	0.193					1.10	0.35	3.50	0.866				
	VmeanX	Q1 (best)	Reference				0.57	0.48	0.66	0.117	Reference				0.62	0.53	0.71	0.009
		Q2 (good)	1.75	0.52	5.87	0.368					1.44	0.41	5.07	0.567				
		Q3 (fair)	1.48	0.43	5.12	0.534					1.11	0.29	4.21	0.877				
		Q4 (poor, failed)	2.34	0.84	6.55	0.104					1.39	0.41	4.76	0.602				
	VmeanY	Q1 (best)	Reference				0.54	0.45	0.63	0.368	Reference				0.62	0.53	0.70	0.013
		Q2 (good)	0.85	0.28	2.61	0.775					0.80	0.25	2.50	0.695				
		Q3 (fair)	0.71	0.22	2.26	0.558					0.60	0.18	2.02	0.411				
		Q4 (poor, failed)	1.28	0.53	3.10	0.584					0.75	0.26	2.18	0.598				

Bold values significant *p*-value < 0.05

^a^Male = Reference

For the adjusted models, Romberg StdElA was found to be the one with the highest AUC (0.67, CI 0.59–0.75, *p* < 0.001). Although most of the models were not statistically significant, all adjusted models showed higher and significant AUCs than the crude models (Table 6).

## Discussion

Balance and postural control play a major role in maintaining independence and mobility, and are considered strong predictors of falls [31], both in healthy subjects [2, 38] and in patients with RA [9, 12]. The results of this study indicate that higher age, higher score in HAQ, low FICSIT-4 score, and a failed one-leg stand are independently associated with prospective falls. Moreover, the sway parameters medio-lateral sway of the Romberg and semitandem stances, the area of sway of the semitandem stance, the total path length and velocity of sway of the semitandem and tandem stances could also be used to predict falls. A discussion of the associations of age and HAQ with falls have been reported elsewhere [10]. The following section focuses on the interpretation of the balance measures.

The physical performance measures FICSIT-4, SPPB and the Romberg test were used to assess standing balance. Group comparisons revealed significant differences between the falls group and the non-fallers for FICSIT-4 and failed one-leg stand. Moreover, in the univariate logistic regression analysis, the poor FICSIT-4 score category (0–4) and one-leg standing as a single test showed significant associations with falls, which was not the case for the SPPB. Gait speed and CRT as independent variables and components of the SPPB also showed no significant associations (shown elsewhere [10]). The remaining difference, in terms of balance assessment between FICSIT-4 and SPPB, was the performance of a one-leg stance in the FICSIT-4. Consequently, it can be assumed that the one-leg stand is the most discriminating factor between fallers and non-fallers. This result is in line with the systematic review by Brenton-Rule et al. [7]. In the review they compared, among other things, the predictive power of different balance tests on falls of patients with RA. Included were studies in which the balance with various tests was measured, such as the duration of one-leg stance, the Romberg test, postural sway or composite scores, e.g. the Tinetti balance test. A significant risk was demonstrated to exist for the case reduced duration of one-leg stance and incomplete Romberg tests. In a meta-analysis of longitudinal cohort studies, it was investigated which measurements for predicting independence in activities of daily life (ADL) in persons older than 65 years yielded the best results [39]. The study’s authors concluded that a reduced duration of a one-leg stand was associated with poorer results in the ADLs and thus, an increased risk of falling, which is in agreement with the results of the present study.

Posturography results of the present study showed a higher proportion of fallers in the worst quartile (Q4) of the postural sway variables compared to the reference quartile (Q1). Numerous sway variables of the Romberg, semitandem and tandem stance positions showed significant group differences between fallers and non-fallers. The medio-lateral sway of both path length and velocity showed significant associations with falls in the Romberg and semitandem stance. In a prospective study by Stel et al. involving a sample of an older population the medio-lateral sway during normal standing (Romberg) was evaluated and it was found to be strongly associated with recurrent falls [40]. This finding was also reported for a systematic review of Piirtola and Era [23]. Melzer et al. [41] observed that medio-lateral sway of narrow stands of the semitandem or tandem stances can be used to discriminate between fallers and non-fallers. In a previous study by Wiegmann et al., it was also observed that with a higher degree of difficulty of the balance position and with reduced postural control, the medio-lateral sway increases [36]. Comparisons between healthy persons and patients with rheumatic diseases have shown, that if patients have higher medio-lateral sway they have reduced postural control [42–44].

No associations were found for anterior–posterior sway in the logistic regressions. Only the unadjusted logistic regression model of anterior–posterior sway in the tandem position showed a significant and high AUC, such that it can be probably used to discriminate between falls and no falls, but the ORs were not significant. There are studies in which it has been shown that there is higher anterior–posterior sway in patients with RA compared to healthy subjects [43, 45, 46], but to the best of the authors knowledge there are no studies containing reports about its impact on fall risk in patients with RA. However, higher sway generally indicates less postural control. Thus, patients with RA have poorer balance control in medio-lateral as well as in anterior–posterior direction compared to healthy subjects [42–46].

The area of sway in semitandem, the total path length and velocity of sway in semitandem and tandem were associated with falls in the unadjusted models. The results of a study by Kawabata et al. [11] confirmed that the path length of semitandem and tandem can be used to discriminate between fallers and non-fallers. However, semitandem had an associated advantage due to the difficulty of the tandem position resulting in too many failures. Consequently, as Kawabata et al. [11] highlighted, it is important to fit the most appropriate assessment to the individual's level of functioning. The results of their work indicated that the higher the degree of difficulty, the higher the proportion of subjects who failed. For the assessment of sway parameters, where the position should be held for 10 s for an accurate measurement, the position should not be too difficult for the individual to perform accurately, but it must also be sufficiently challenging to reveal balance deficits. According to the data in this work, this appears to be the case for the semitandem and tandem position for patients with RA, with the semitandem being more robust due to the lower failure rate.

In terms of postural control and falls, there is conflicting evidence on the role of disease activity and duration of disease. In some previous studies there has been found to be disease activity associated with increased risk of falls [9], whereas in others this was found not to be the case [12, 14, 33, 47]. Toprak et al. [48] analysed the sway velocity during a one-leg stance in a sample of patients with RA compared to healthy subjects. They found significant correlations between sway velocity and disease activity, such as swollen joints and DAS28 [48]. In contrast, in a study by Ekdahl et al. it could not be confirmed that RA disease characteristics are relevant for explaining postural control [49]. According to Böhler et al., one possible reason could be that disease activity was measured at the baseline in this study rather than at the time of the fall or at the end of the follow-up period [50]. An analysis of a sample group with high disease activity or a subgroup of recurrent fallers might have produced different results [51].

However, a longer RA disease duration is associated with higher HAQ scores [52]. In the current study, fallers showed higher restrictions in ADLs and a reduced balance confidence. HAQ was shown to have a strong association with falls, which has been confirmed in previous prospective studies [9, 33]. The mean score of the ABC-scale of both groups in the present work was > 80%, thus indicating a high level of balance confidence [29]. Fall history and fear of falling can affect balance confidence, self-reported physical activity and mobility in daily life and fall risk [7]. Previous falls for this study cohort were reported elsewhere [10], but there were no significant differences observed between falls group and non-fallers. Disease activity and systemic changes due to RA inflammation have an impact on muscle strength, joint deformation, pain and individual mobility [7], thus can affect balance confidence and ADLs. In the sample investigated in this work, the perceived limitations in ADLs did not seem to have influence on the balance confidence, possibly due to low disease activity within the group. Nevertheless, strengthening balance confidence and reducing the fear of falling remain important aspects of fall prevention.

### Strength and limitations

This study has multiple strengths, including the prospective design and the use of quarterly fall diaries, which made data collection more reliable than retrospective studies reporting on fall history. The regular telephone contacts and reminders by the study centre made it possible to record falls promptly and keep the drop-out rate low. One source of weakness in this study, which could have affected the results, was the incidence of falls in the study group. Previously reported findings on the incidence of falls in patients with RA suggest that the incidence of 20.2% of this study is a relatively low rate [10, 13]. Furthermore, a low disease activity, and a “young” sample with a mean age of 60.2 years and a wide range of ages might be the cause of bias in terms of a reduced incidence of falls in the sample. One reason for this could be that in the original study the prevalence of sarcopenia was explored rather than the risk of falls. The one-year follow-up was made possible by further funding and, was planned and carried out throughout the research process. For this reason, other risk factors that have a significant influence on balance, such as medication, comorbidities and foot deformities [7], were not covered in the baseline. Further, according to Bouchaala et al. [53], balance performance varies during the time of the day and culminates in the afternoon at 2 pm. For logistical reasons, however, this influencing factor could not be taken into account because the subjects were invited to the baseline assessment throughout the day. It was also not systematically investigated at what time a fall occurred.

During the execution of the Romberg test a high failure rate was seen for the tandem stand onwards, which is problematic especially for research due to the missing values. For the statistical analysis in the current study, the issue could be solved by constructing quartile variables for the sway parameters and by integrating dichotomous variables, e.g. “One-leg _Balance (failed/done)”. In clinical practice, missing values can be ignored and, in particular, a single one-leg stand, if failed, seems to be a valid tool for predicting fall risk; in the context of research the FICSIT-4 is superior to the Romberg tests, because the composite score integrates failed positions.

## Conclusions

The results of the presented analyses support the use of the FICSIT-4 assessment, including the ability to perform a one-leg stand, to determine fall risk in rheumatoid patients. For the screening of fall risk in clinical practice a one-leg stand might be appropriate, as this can be used to differentiate between patients at high risk of falling (failed) and those at low risk of falling (held for 10 s).

For the assessment of sway parameters in a population of RA patients, the semitandem stand showed the most applicable results. In order to determine fall risk in this population, path length, area of sway, velocity of sway and medio-lateral sway during a semitandem stand can be used. The semitandem stand was safe to perform, was completed by almost all subjects and was moderately challenging for the postural system. This position is particularly suitable for samples over a wide range of age as well as for the elderly or subjects with a chronic disease. For younger and active subjects, positions should be chosen that challenge the balance system to the maximum. This can be achieved by using one-leg stands, closed eyes or dynamic positions.

For the development of prevention or physical therapy programmes, the training of medio-lateral and anterior–posterior balance strategies should be focused on. The mobility and perception of the foot, and the prevention of joint deformities in order to improve balance ability should be prioritised.

## Data Availability

The datasets used and/or analysed during the current study are available from the corresponding author on reasonable request.

## Abbreviations

ABC-scale: Activity-specific Balance Confidence Scale
AUC: Area under the curve
CoP: Centre of pressure
CRP: C-reactive protein
DAS28_CRP_: Disease Activity Score with CRP
FICSIT-4: Frailty and Injuries: Cooperative Studies of Intervention Technique-4
FIR Filter: Finite Impulse Response Filter
HAQ: Health Assessment Questionnaire
RA: Rheumatoid Arthritis
ROC: Receiver-operating characteristics
SPPB: Short Physical Performance Battery
